# A Functional Approach to Deconvolve Dynamic Neuroimaging Data

**DOI:** 10.1080/01621459.2015.1060241

**Published:** 2016-05-05

**Authors:** Ci-Ren Jiang, John A. D. Aston, Jane-Ling Wang

**Keywords:** Compartmental modeling, Functional response model, Kinetic modeling, Neuroimaging.

## Abstract

Positron emission tomography (PET) is an imaging technique which can be used to investigate chemical changes in human biological processes such as cancer development or neurochemical reactions. Most dynamic PET scans are currently analyzed based on the assumption that linear first-order kinetics can be used to adequately describe the system under observation. However, there has recently been strong evidence that this is not the case. To provide an analysis of PET data which is free from this compartmental assumption, we propose a nonparametric deconvolution and analysis model for dynamic PET data based on functional principal component analysis. This yields flexibility in the possible deconvolved functions while still performing well when a linear compartmental model setup is the true data generating mechanism. As the deconvolution needs to be performed on only a relative small number of basis functions rather than voxel by voxel in the entire three-dimensional volume, the methodology is both robust to typical brain imaging noise levels while also being computationally efficient. The new methodology is investigated through simulations in both one-dimensional functions and 2D images and also applied to a neuroimaging study whose goal is the quantification of opioid receptor concentration in the brain.

## 1. INTRODUCTION

Positron emission tomography (PET) is an in vivo neuroimaging technique for studying biological processes in humans. It is almost unique among the major neuroimaging modalities, in that it can be used to study neurochemical concentrations and associated changes in a quantifiable way. PET works on the principle of using an injected radioactive tracer compound specifically designed for the biological process of interest and tracking its presence throughout the target organ through the emitted radiation of the radioactively decaying compound. It is a quantitative technique, as opposed to say functional magnetic resonance imaging (fMRI), in that the amount of radiochemical injected can be used to establish the concentrations present in the target organs. This has led it to be almost universally used in the diagnosis of certain cancers through fluorodeoxyglucose (FDG) PET scans, which as a surrogate for glucose, can be used to target tissues with high metabolic rates, such as tissues containing cancer cells. Indeed, it is not only used for cancer diagnosis and localization in the brain, but also throughout the body (Gambhir 2002; Hsieh 2012).

In addition to diagnostic and clinical usage, PET also can be used to investigate neurochemical processes to help further understanding of the brain. Individual neurochemical transmitter systems can be targeted through the design of radiotracers that mimic the behavior of these chemicals. As might be imagined, this involves considerable complex radiochemistry to design suitable radiolabeled tracers which can be detected by the PET camera. However, there are now many tracers available to target systems in addition to metabolism such as the dopamine system (Wagner et al. 1983), the serotonergic system (Drevets et al. 1999), and the opioid receptor system (Jones et al. 2004). Indeed, it is the last of these, the opioid system, that is the motivation for this work. The opioid system controls the brain's reaction to pain (Pasternak 1993), and has been associated with a number of conditions and diseases including changes in emotional responses (Filliol et al. 2000), addiction (Wise 1996), and Alzheimer's disease (Jansen et al. 1990). The data which will be analyzed later in this article is taken from part of a large study on the role of the opioid system in epilepsy. It is of great interest to get accurate quantifiable estimates of opioid receptor concentrations and densities throughout the brain in normal subjects as a precursor to understanding the role of receptor changes in disease diagnosis, prognosis, and treatment.

PET scans, in a similar way to fMRI scans, consist of three-dimensional volumes of data recorded over time, leading to large datasets with time courses from millions of spatial locations (voxels). The time courses associated with PET data are characteristically nonlinear in that, being associated with chemical reactions, they are routinely modeled as coming from ordinary differential equation (ODE) systems, where first-order linear kinetics can be used to model the data (Gunn, Gunn, and Cunningham 2001). These kinetics are routinely associated with compartmental models, which consist of abstract compartments within each voxel. The transfer of material from one compartment to another is assumed to follow a first-order ODE. For more information on compartmental models see Godfrey (1983). However, there is increasing evidence both from biological experiments and statistical analysis that such models are not adequate for the data (O'Sullivan et al. 2009), not least because each voxel represents an inhomogeneous mixture of cells leading to a mixture of compartmental processes (assuming the compartmental assumption is even made). Therefore, compartmental analysis can produce both biased and incomparable estimates across the brain. In addition, fitting methods which are stable for large numbers of voxels, such as nonnegative least squares, tend to have parameter dependent bias, while methods such as nonlinear least squares tend to be somewhat unstable (Peng et al. 2008).

To account for the model discrepancies while still maintaining a robust approach to model fitting, this article explores a nonparametric deconvolution model for PET analysis. The input (through the blood flow to the brain) can be measured online to all extents and purposes continuously with virtually no measurement error relative to the error in the measured voxelwise PET data, as the sampling on the measured radioactivity of the blood is done outside the body using a sensitive blood monitor via an arterial canula to produce a smooth continuous input curve (Lammertsma et al. 1991). This allows one of the functions in the deconvolution to be known (i.e., this is not a blind deconvolution problem), but the inherent difficulties of deconvolving the noisy measured output function are all still present. We present a new methodology for deconvolution and analysis of data. This new methodology works when there are multiple observations of the convolved functions, and can also be used when the functions are possibly dependent on a covariate.

The analysis is based on using functional principal component analysis (FPCA). Our methodology involves a presmoothing step to reconstruct the image, followed by a deconvolution step to recover the impulse response function. Presmoothing decreases the noise in the data, hence can reduce overall potential biases in further analysis, as even though smoothing itself introduces a small bias, in many nonlinear situations, parameter or functional biases can be noise level dependent. In parametric nonlinear models such as compartmental models, this is well known (Peng et al. 2008), while here the errors in the observed functions are somewhat similar to those in measurement error models, which yield biases in traditional regression analysis. The presmoothing also produces functions that are smoother than the original data, making subsequent deconvolution easier, as large independent measurement errors tend to result in considerable instability in deconvolution settings.

As for the deconvolution approach, ours differs from traditional deconvolution methods and has inherent computational advantages in that we treat the sample of dynamic PET data on all voxels as functional data, and apply FPCA to reduce the dimension of the data, so that the deconvolution only needs to be performed on the mean and eigenfunctions of the data. This has substantial computational advantages as while there are millions of spatial voxel locations, often only a few basis functions in the FPCA basis are needed to adequately describe the temporal curves, requiring only a very small number of actual deconvolutions to be performed. Moreover, it is not the actual deconvolutions that are the focus of the PET study. Of primary interest in many PET studies is the volume of distribution, *V_T_*, the integral of the impulse response function of the system at each voxel. Under various biological assumptions, *V_T_* can be used to determine the receptor density of the underlying neurotransmitter (Innis et al. 2007). As advocated by O'Sullivan et al. (2009), this will be approximated by the integral of the deconvolved response function generated from the observed data, which in itself is a more meaningful measure as it is less dependent on the particular compartmental model fit assumed.

The article proceeds as follows. In the next section, the moderately general methodology, inspired by PET data, is introduced for deconvolution of multiply observed functions through the use of FPCA. In Section 3, the methods are assessed through simulation, not only on 1D functions, but also on moderately realistic 2D image slices where both spatial correlations and nonhomogeneous noise models, typical of those found in PET studies, are used. In Section 4, the methods are applied to measured [^11^C]-diprenorphine scans taken from healthy volunteers and are used to provide voxelwise quantification of receptor concentration without resorting to compartmental assumptions. The final section discusses some of the possible extensions of this work.

## 2. METHODOLOGY

Let *C_i_*(*t*) be the concentration curve of voxel *i* in PET analysis, where *i* is a generic index representing a spatial location. The conventional assumption is that

(1) $$\begin{array}{c} C_{i}\left(t\right)=\left(I⊗M_{i}\right)\left(t\right)=\int_{0}^{t}I\left(t-s\right)M_{i}\left(s\right)ds, \end{array}$$

where *I*(*t*) is a known input function and *M_i_*(*t*) is the unknown impulse response function (IRF) of voxel *i*. We assume that the input function *I*(*t*) is smooth and positive over the entire range of the integration. This is true in practice given the nature of the input function being the amount of tracer in the blood plasma. In reality, *C_i_*(*t*) is not observed, but rather, a noise contaminated version of *C_i_*(*t*)exp ( − λ*t*) is observed (Aston et al. 2000) at discrete time points, *t* = *t*_1_, …, *t_p_* where λ is the known decay constant of the radioisotope (in the case of ^11^ *C*, this is 5.663 × 10^− 4^ *s* ^− 1^.). Suppose there are *n* voxels and *p* observations per voxel. Hence, the observations for the *i*th voxel are *Y_ij_* = *X_i_*(*t_j_*) + ϵ_*ij*_, where *X_i_*(*t_j_*) = *C_i_*(*t_j_*)exp ( − λ*t_j_*) and ϵ_*ij*_ are independent noise for *i* = 1, …, *n* and *j* = 1, …, *p*. Here, the independence assumption on the errors in time can be largely justified on the basis of the independent Poisson decay nature of radioactivity (Carson and Lange 1985), while the implications of assuming spatial independence will be discussed later.

The goal of PET analysis is to estimate the volume of distribution (*V_T_*) at each voxel *i*, which is *V_T_*(*i*) = ∫^τ^_0_ *M_i_*(*t*)*dt*, where τ is the end of the experimental time (and is typically taken to be infinity in parametric modeling). To estimate *V_T_*, it is necessary to estimate the IRF *M_i_*(*t*) through deconvolution. As we are using a nonparametric estimator in the deconvolution, it is not possible to extrapolate the *V_T_* to infinity (as this would require a parametric model), but this finite truncated version could well be preferred in many situations (O'Sullivan et al. 2009), particularly given the known difficulties of function extrapolation.

### 2.1 Spatial Curve Pre-Regularization

With the presence of noise in the output data *Y_ij_*, our first step is to reconstruct *X_i_*(*t*) for all voxels. Instead of handling these temporal curves voxel by voxel, we borrow spatial information from all voxels by applying a spatially adapted smoother to *Y_ij_* across all time points (*t*) and spatial/voxel locations, denoted as $Z_{i}$ for the *i*th voxel. Depending on the dimension of the image, a three (for 2D images) or four (for 3D images) dimensional smoother is used to reconstruct the latent signals. For the PET data in Section 4, $Z_{i}$ is three-dimensional, so a four-dimensional smoother is employed. This may seem a formidable task, given the large amount of available data (32 time points and 150,784 brain voxels), but it is feasible if one adopts an computationally efficient approach. For those who are interested in the theoretical parts of this step, the following are the specific assumptions we make. We assume that the orders of bandwidths are all of the same order as *h*. We also assume that the second derivatives of *X_i_*(*t*), the variable bandwidth function *h_T_*(*t*), formally defined in Section 2.2, and the variance of *X_i_*(*t*) are continuous and bounded. For a *k*-dimensional smoother, *h* → 0 and *nph^k^* → ∞.

Let ${\hat{X}}_{i}\left(t\right)$ be the smoothed estimate of *X_i_*(*t*). Specifically,

(2) $$\begin{array}{ccc} {\hat{X}}_{i}\left(t\right) & = & {\hat{b}}_{i,0}, \end{array}$$

where

b^i= arg  min (bi0,...,bi4)∑k=1n∑j=1pKkj,h(zi,t)×Ykj-bi,0-∑ℓ=13bi,ℓ(ziℓ-zkℓ)-bi,4(t-tj)2,

$K_{kj,h}\left(z_{i},t\right)=\frac{1}{\beta {\hat{h}}_{T}\left(t\right)h_{z_{1}}h_{z_{2}}h_{z_{3}}}K\left(\frac{z_{i1}-z_{k1}}{h_{z_{1}}},\frac{z_{i2}-z_{k2}}{h_{z_{2}}},\frac{z_{i3}-z_{k3}}{h_{z_{3}}},\frac{t-t_{j}}{\beta {\hat{h}}_{T}\left(t\right)}\right)$ is a four-dimensional kernel function (an Epanechnikov kernel was used in the data analysis), $z_{i}$ is the spatial location for voxel *i*, *h*'s are the bandwidths in the spatial coordinates, ${\hat{h}}_{T}\left(t\right)$ is the variable bandwidth, and β is the calibration coefficient for ${\hat{h}}_{T}\left(t\right)$. The kernel function *K* is assumed to be a symmetric probability density function with bounded support. Note that constant bandwidths are employed for spatial coordinates (in the application, one bandwidth is chosen for all three dimensions), but an adaptive local bandwidth for the time dimension is applied (see Section 2.2 for details). The reconstructed concentration function for *C_i_*(*t*) is

(3) $$\begin{array}{c} {\hat{C}}_{i}\left(t\right)={\hat{X}}_{i}\left(t\right)\exp\left(\lambda t\right). \end{array}$$

If a kernel estimator is chosen, a product kernel can be applied to save computational time, which is equivalent to smoothing each coordinate of time and space sequentially.

### 2.2 Variable Bandwidth

In most PET analysis, particularly in the spatial domain, smoothing is based on heuristic assessments determined by the individual researcher. Here, we propose to use data-driven methods to select the bandwidth choices. A constant bandwidth is suitable for the spatial coordinates as the covariance structure, while subtly changing across the image, does not vary substantially. However, in the time coordinate, due to the denser measurements at the beginning of the time period and the sharp peak near the left boundary, a nonconstant bandwidth is required. To retain the peak without compromising the performance at other temporal locations, a locally adaptive bandwidth function is recommended and applied in our analysis. Essentially, a smaller bandwidth is preferred near the peak location, while larger bandwidths are used near the right boundary, where the curve is relatively flat. This is also consistent with the fact that the noise in PET data can be crudely seen as being Poisson distributed due to the radio labeled nature of the data (Carson and Lange 1985).

**Figure 1:**
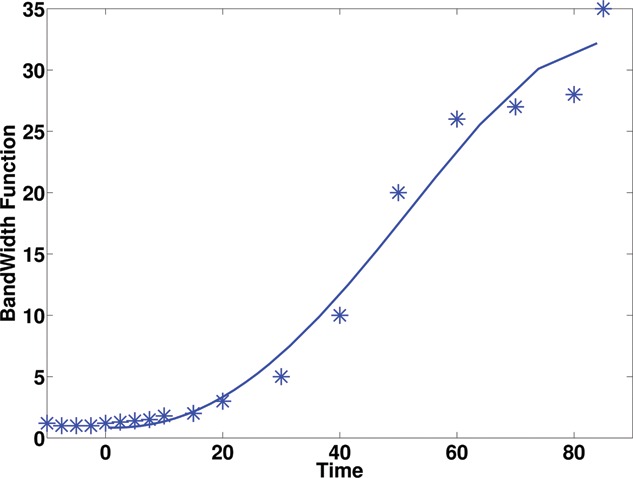
The resulting locally adaptive bandwidth for PET time-course data.

We undertook the following pragmatic approach to design such a bandwidth function. First, a number of time locations (*n_b_*) $(t_{\left(1\right)},...,t_{\left(n_{b}\right)}),$ where the time-course data were observed, were selected (we used *n_b_* = 13 in the application, which was approximately 1/3 of the time points in the time course). At each location, the bandwidth *h_T_*(*t*) at location *t* was chosen such that the interval [*t* − *h_T_*(*t*), *t* + *h_T_*(*t*)] contains at least four observations. Further, boundary correction was employed to ensure the resulting bandwidth function was positive when *t* was close to zero. A fourth-order polynomial was applied to the pair set {(*h_T_*(*t*_(*i*)_), *t*_(*i*)_)|*i* = 1, …, *n_b_*} to obtain a smooth bandwidth function. The resulting bandwidth function ${\hat{h}}_{T}\left(t\right)$ (shown in Figure 1) was further multiplied by a constant β. The constant β serves to facilitate calibration of the final local bandwidths, because the choice of local bandwidths for *h_T_*(*t*) was subjective, and thus β, which was determined by cross-validation, allowed this subjective choice to be adapted to the data. This form of bandwidth selection has been shown to work well in previous studies on smoothing prior to parametric compartmental modeling (Jiang, Aston, and Wang 2009).

While several tuning parameters need to be chosen for the analysis, this is not uncommon in PET, as data is usually smoothed to increase the signal-to-noise ratio or to facilitate population studies. However, as mentioned above, most analyses use the default settings of whatever software package is being used, while we here prefer to determine a good choice of bandwidth through cross-validation.

For every bandwidth candidate, each time, we randomly remove *n_cv_* voxels, use the observations of the remaining *n* − *n_cv_* voxels to estimate the mean functions of the removed voxels. This procedure repeats *N* times and we choose the bandwidth minimizing the mean squared errors between fitted curves and observations. We set *n_cv_* = 1 and *N* = 5000 in our data analysis. The bandwidths for space and time are selected sequentially to save computation time.

### 2.3 Deconvolution Based on FPCA

With the concentration curve reconstructed for each voxel, one can perform deconvolution voxelwise to recover the IRF. However, attempting to perform automated deconvolution over such a large number of functions is inherently problematic and computationally costly. Alternatively, we take the viewpoint that the concentration curves are random curves, a.k.a. functional data (Ramsay and Silverman 2005), so a functional approach can be employed to model these curves. Since convolution is a linear operator, it is advantageous to adopt a linear mixed-effects model approach to represent these functional data. Since we do not assume that the shapes of the IRFs are known a priori, a nonparametric basis function is the preferred choice and we adopt parsimonious basis functions through principal component analysis.

Principal component analysis is a popular dimension reduction approach for multivariate data and has been extended to functional data that are in the form of random curves and termed FPCA. Many different FPCA approaches have been developed, such as by Dauxois, Pousse, and Romain (1982), Rice and Silverman (1991), Boente and Fraiman (2000), Cardot (2000), and Yao, Müller, and Wang (2005). We adopt a similar approach as Yao, Müller, and Wang (2005), but with a slightly different model that was advocated in Jiang, Aston, and Wang (2009) for PET time course data. Specifically, a multiplicative random effects model was proposed there, motivated by the likely randomness in chemical rates (e.g., induced by spatially varying neurochemical receptor densities) leading to multiplicative changes in the curves. Thus, we adopt the following modified Karhunen–Loève decomposition of *C_i_*(*t*), which includes an additional random effect term *A*_*i*0_ on the mean function:

(4) $$\begin{array}{c} C_{i}\left(t\right)=A_{i0}\mu \left(t\right)+\sum_{k}A_{ik}\varphi_{k}\left(t\right), \end{array}$$

where μ(*t*) = *E*{*C_i_*(*t*)} is the mean function, *E*(*A*_*i*0_) = 1, φ_*k*_(*t*) are the eigenfunctions of the covariance of *C_i_*(*t*) − *A*_*i*0_μ(*t*) with the corresponding non-increasing eigenvalue ζ_*k*_, and *A_ik_* is the *k*th functional principal component score.

In general, deconvolution is an ill-posed problem. However, due to the positivity of the input function *I*(*t*), (2.1) and (2.4) imply that

(5) $$\begin{array}{c} M_{i}\left(t\right)=A_{i0}\mu^{d}\left(t\right)+\sum_{k}A_{ik}\varphi_{k}^{d}\left(t\right), \end{array}$$

where μ(*t*) = (*I*⊗μ^*d*^)(*t*) and φ_*k*_(*t*) = (*I*⊗φ^*d*^_*k*_)(*t*). Therefore, deconvolution only has to be performed on the mean function and the likely small number of eigenfunctions needed to give a good representation of the data. This has considerable computational savings compared to performing it on hundreds of thousands of spatial voxels and is one of the main advantages of our approach. It should, however, be noted at this point that φ^*d*^_*k*_(*t*) do not necessarily form an eigendecomposition of *M_i_*(*t*) (as deconvolution does not necessarily preserve orthogonality) but are rather a basis of the deconvolved space.

To perform the deconvolution, we consider the following strategy, which will be illustrated on μ(*t*). Suppose that μ(*t*) or an estimate of it is available at times *s*_0_, *s*_1_, …, *s_m_*, where *s*_0_ = 0 and *s_m_* = τ. Let ***μ*** ^*T*^ = (μ(*s*_1_), …, μ(*s_m_*)). When *m* is large, $\mu \approx A\mu^{d},$ where

(6) $$\begin{array}{c} A=\left(\begin{array}{ccccc} I\left(s_{1}\right)\frac{s_{1}}{2} & 0 & 0 & ... & 0\\ I\left(s_{2}\right)\frac{s_{1}}{2} & I\left(s_{2}-s_{1}\right)\frac{s_{2}}{2} & 0 & ... & 0\\ \vdots & \vdots & \ddots & \vdots \\ I\left(s_{m}\right)\frac{s_{1}}{2} & I\left(s_{m}-s_{1}\right)\frac{s_{2}}{2} & I\left(s_{m}-s_{2}\right)\frac{s_{3}-s_{1}}{2} & \vdots & I\left(s_{m}-s_{m-1}\right)\frac{s_{m}-s_{m-2}}{2} \end{array}\right),\\ \;(2.6)\; \end{array}$$

and ***μ*** ^*d*^ = (μ^*d*^(*s*_0_), …, μ^*d*^(*s*_*m* − 1_))^*T*^. The matrix A can be seen as a linear discretization of the convolution integral. Therefore, we can obtain an estimate of ***μ*** ^*d*^ by

(7) μd^= arg  min μd∥μ-Aμd∥2.

This allows the deconvolution procedure to be framed as a linear regression problem, allowing the use of the usual standard least squares formulation. In the measured data analysis and simulations in the next sections, we interpolated the smoothed PET time courses to *m* = 250 to balance computational complexity with discretization error.

This is of course not the only possible deconvolution strategy that could be used, and many others exist in the literature, including spline-based deconvolution as used by O'Sullivan et al. (2009, 2014). However, it is very simple and computationally efficient to implement, and as will be seen in the simulations produces reasonable estimates of the deconvolved curves.

### 2.4 Estimation of FPCA

Since the mean function μ(*t*) and eigenfunctions φ_*k*_(*t*) associated with the concentration function *C_i_*(*t*) are unknown, they need to be estimated first before one can implement the deconvolution in (2.7).

*Estimation of the mean function* **μ(t).** One could use the mean function of the reconstructed ${\hat{C}}_{i}\left(t\right)$ in (2.3). However, as ${\hat{C}}_{i}\left(t\right)$ results from smoothing, the bias inherited at this step in the reconstruction leads to a biased estimate of μ (this is particularly affected by the smoothing being later combined with the decay correction, which is an exponential transform). We thus estimate μ through the sample mean of *Y_ij_*. Let $Y_{\cdot j}=\frac{1}{n}\sum_{i=1}^{n}Y_{ij}$ be the cross-sectional mean (without any smoothing) of the observed data *Y_ij_* at time *t_j_*. The estimate for μ(*t*) is

(8) $$\begin{array}{ccc} \hat{\mu }\left(t\right) & = & Y_{\cdot j}\exp\left(\lambda t\right),\;\;\text{for}\;t=t_{j},\\ & = & \text{the}\;\text{linear}\;\;\text{interpolated}\;\;\text{value}\;\;\text{of}\;\hat{\mu }\left(t_{k}\right)\;\text{and}\;\hat{\mu }\left(t_{k+1}\right),\\ & & \;\text{for}\;t_{k}<t<t_{k+1}. \end{array}$$

The resulting linearly interpolated estimate $\hat{\mu }\left(t\right)$ is unbiased at *t* = *t_j_* for all *j* = 1, …, *p*, and has a smaller bias at other *t* than the mean of ${\hat{C}}_{i}\left(t\right)$. Of course, other interpolating schemes such as cubic spline interpolation could be substituted at this point, but linear interpolation is faster and seems to work well for PET data.

*Estimation of *A*_*i*0_.* The estimate of the multiplicative coefficient at voxel *i* is

(9) $$\begin{array}{c} {\hat{A}}_{i0}=\int_{0}^{\tau }{\hat{C}}_{i}\left(t\right)\hat{\mu }\left(t\right)dt/\int_{0}^{\tau }{\left(\hat{\mu }\left(t\right)\right)}^{2}dt, \end{array}$$

where $\hat{\mu }\left(t\right)$ is the estimate of the mean function μ from (2.8) and ${\hat{C}}_{i}\left(t\right)$ is from (2.3). It should, of course, be noted here that the resulting *A*_*i*0_ will not necessarily have the usual property of having expectation one. This results from estimating the *A*_*i*0_ from the smoothed data, while the mean function is derived from the unsmoothed data. However, in practice the difference between the two is small, and considerably less variable estimation results from using smoothed data to estimate *A*_*i*0_ (a classic bias-variance trade-off).

*Estimation of the eigenfunctions **φ_k_** and* *principal component scores.* We estimate the covariance function by the sample covariance of ${\hat{C}}_{i}\left(t_{j}\right)-{\hat{A}}_{i0}\hat{\mu }\left(t_{j}\right),$ where ${\hat{C}}_{i}$ is from (2.3), ${\hat{A}}_{i0}$ is from (2.9), and $\hat{\mu }$ is from (2.8). Specifically,

(10) $$\begin{array}{ccc} \hat{\Gamma }\left(t_{j},t_{k}\right) & = & \frac{1}{n}\sum_{i=1}^{n}\left\{{\hat{C}}_{i}\left(t_{j}\right)-{\hat{A}}_{i0}\hat{\mu }\left(t_{j}\right)\right\}\\ & & \times \{{\hat{C}}_{i}\left(t_{k}\right)-{\hat{A}}_{i0}\hat{\mu }\left(t_{k}\right)\} \end{array}$$

for 1 ⩽ *j*, *k* ⩽ *p*. Once the covariance is obtained, the eigenfunctions can be estimated by solving the eigen-equations at a dense grid. Let ${\hat{\varphi }}_{k}\left(t\right)$ be the estimate of φ_*k*_(*t*), The principal component scores *A_ik_* can be estimated by

(11) $$\begin{array}{c} {\hat{A}}_{ik}=\int_{0}^{\tau }\left\{{\hat{C}}_{i}\left(t\right)-{\hat{A}}_{i0}\hat{\mu }\left(t\right)\right\}\;{\hat{\varphi }}_{k}\left(t\right)\;dt. \end{array}$$

*Number of components.* The number of eigenfunctions *L* for voxel *i* is selected by

(12) $$\begin{array}{c} R^{2}\left(i,L\right)=1-\frac{\text{var}\left\{Y_{i}\left(t\right)-{\hat{C}}_{i}\left(t,L\right)\exp\left(-\lambda t\right)\right\}}{\text{var}\{Y_{i}\left(t\right)\}}, \end{array}$$

where ${\hat{C}}_{i}\left(t,L\right)={\hat{A}}_{i0}\hat{\mu }\left(t\right)+\sum_{k=1}^{L}{\hat{A}}_{ik}{\hat{\varphi }}_{k}\left(t\right).$ The above *R* ^2^ is an ad hoc measure for the goodness of fit, but provides a useful summary of how much additional information is gained by adding a further eigenfunction. For the simulation and data analysis in later sections, we adopted a simple rule to select *L* by setting *L* = *k* when *R* ^2^(*i*, *k* + 1) − *R* ^2^(*i*, *k*) < 0.025.

After the number of components *L* is selected by *R* ^2^, the IRFs can be reconstructed through (2.5) and its associated *V_T_* can be estimated by integration of the IRF. Specifically, at the *i*th voxel, these estimates are

(13) $$\begin{array}{ccc} {\hat{M}}_{i}\left(t,L\right) & = & {\hat{A}}_{i0}{\hat{\mu }}^{d}\left(t\right)+\sum_{k=1}^{L}{\hat{A}}_{ik}{\hat{\varphi }}_{k}^{d}\left(t\right),\\ & & \;\mathrm{and} \end{array}$$

(14) $$\begin{array}{ccc} \hat{V_{T}}\left(i,L\right) & = & {\hat{A}}_{i0}\int_{0}^{\tau }{\hat{\mu }}^{d}\left(t\right)dt+\sum_{k=1}^{L}{\hat{A}}_{ik}\int_{0}^{\tau }{\hat{\varphi }}_{k}^{d}\left(t\right)dt. \end{array}$$

The details of our approach are summarized in Algorithm 1.


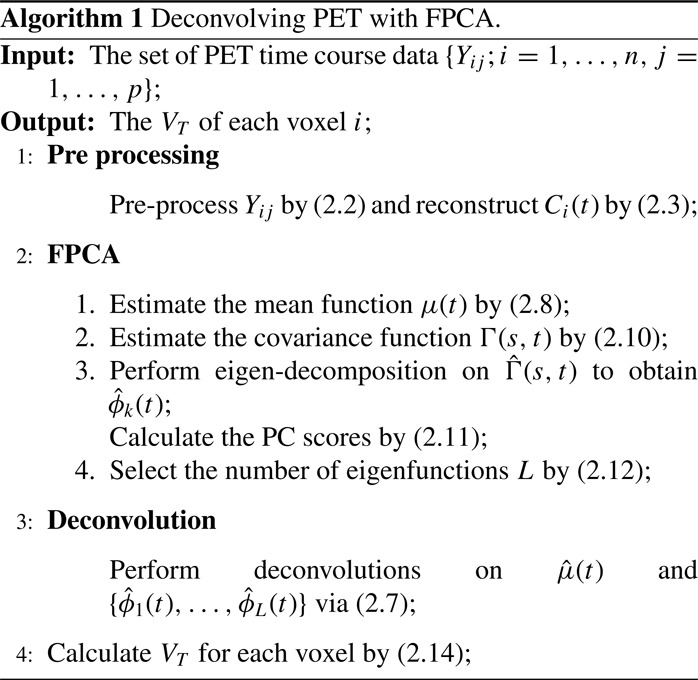


## 3. SIMULATION STUDIES

The proposed methodology is first evaluated on simulated data, both on 1D functions and then in more realistic image settings using 2D image phantoms.

### 3.1 One-Dimensional Function Simulations

Before showing our formal image simulation studies, we would like to demonstrate the use of the deconvolution strategy via FPCA and assess how well it works for general functional data. First, we assume the target functions (IRFs in PET imaging data) can be represented as

$$\begin{array}{c} M_{i}\left(t\right)=\mu_{M}\left(t\right)+\sum_{k=1}^{2}B_{ik}\psi_{k}\left(t\right), \end{array}$$

where *B_ik_* are random variables and ψ_*k*_(*t*) are basis functions. Specifically, we simulate the mean function μ_*M*_(*t*) = 0.0049exp ( − 0.0005*t*) + 0.0018exp ( − 0.0112*t*) and ψ_1_(*t*) = *c*_1_sin (2π*t*/2000) and ψ_2_(*t*) = *c*_2_cos (2π*t*/2000), where *c*_1_ and *c*_2_ are constants which normalize the basis functions in the $L_{2}\text{-}$norm. Specifically,

c1=1∫02000sin2(2πt/2000)dt and c2=1∫02000cos2(2πt/2000)dt.

**Figure 2:**
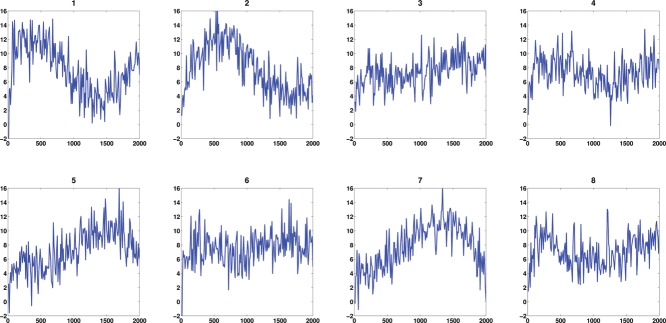
First eight observed curves of the 200 curves in the 1D simulation.

**Figure 3:**
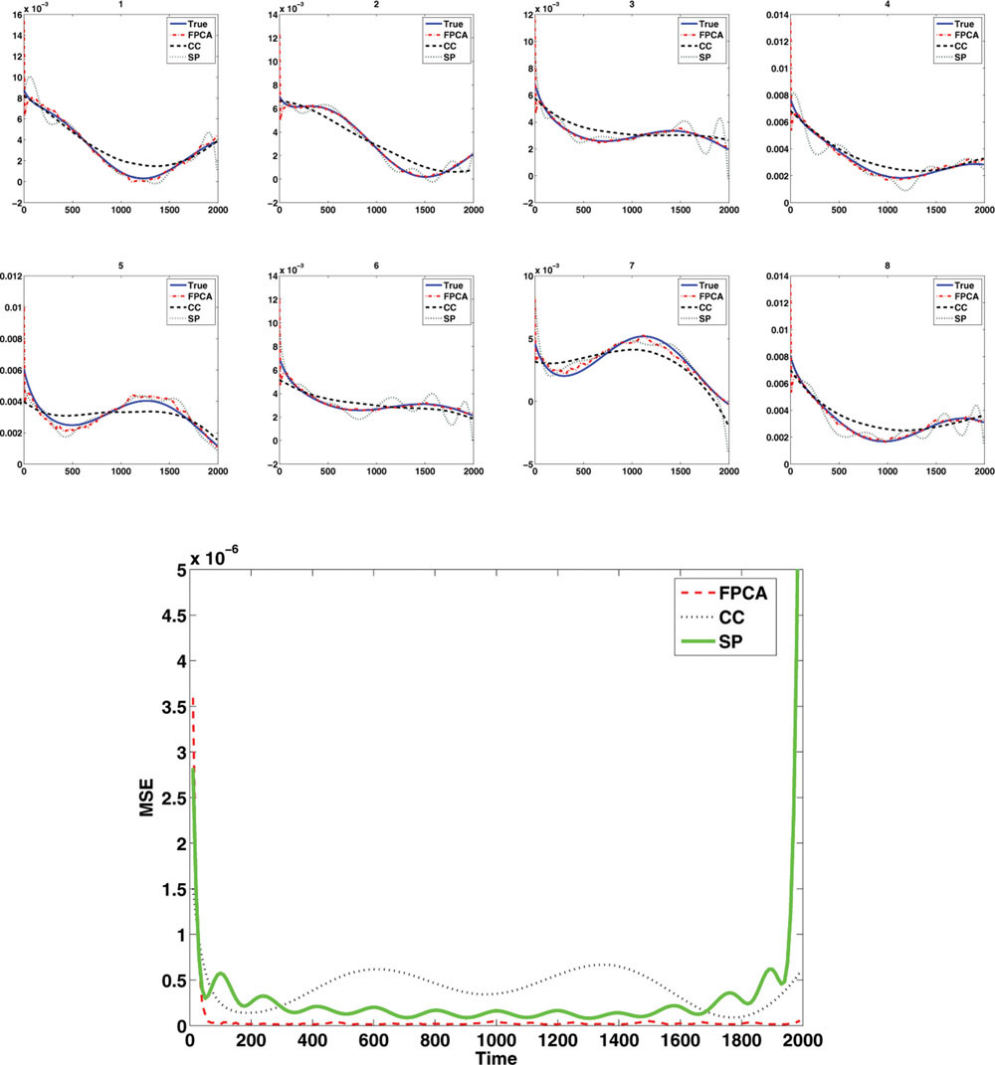
Estimated deconvolved functions and true target function in 1D simulation corresponding to the curves in Figure 2 along with the pointwise MSE for each method.

Also, the random coefficients (*B*_*i*1_ and *B*_*i*2_) for the basis functions are generated from *N*(0, 0.1^2^) and *N*(0, 0.05^2^) respectively. The random functions are then convolved with an arterial input function taken from the [^11^C]-diprenorphine study of the next section truncated at 2000 s. The data observed are further contaminated with independent measurement errors at the observation times,

$$\begin{array}{c} Y_{i}\left(t\right)=\left(I⊗M_{i}\right)\left(t\right)+\varepsilon , \end{array}$$

where ϵ ∼ *N*(0, 2^2^) and where notationally we assume that the errors are only present at the observations, not over the entire continuum. This toy example contains 200 curves with observations made at 200 equally spaced time points and the first eight observed noisy curves are shown in Figure 2. The MATLAB package PACE (Yao, Müller, and Wang 2005) was applied to obtain the mean function and eigenfunctions for the observed functions. Figure 3 indicates that our deconvolution strategy via FPCA performs very well for regular functional data. As the FPCA uses information across all curves, this improves the deconvolution.

**Figure 4:**
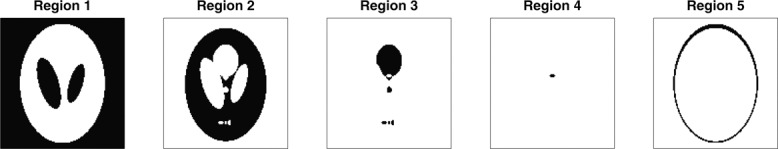
Phantom image: each of the five different regions used in the simulations are indicated.

In addition to the FPCA approach, we compared several other approaches. O'Sullivan et al. (2009) proposed an approach which worked well for region of interest (ROI) data in FDG-PET. It was based on a spline deconvolution of the response function. We implemented an analogous spline based deconvolution (SP), using a weight function suitably chosen for the simulations. We also examined deconvolution as used within the FPCA procedure on a curve-by-curve (CC) basis. However, both the spline deconvolution and the CC deconvolution of the same data is much less accurate (see Figure 3) with a 10-fold increase in mean integrated squared error (MISE) between the FPCA approach and the CC approach (MISE, FPCA: 0.0879 × 10^− 3^, CC: 0.8160 × 10^− 3^, SP: 0.5045 × 10^− 3^), with the spline approach performing slightly better than the CC approach except at the boundaries, but still less well than the FPCA approach. Similar results (not shown) were obtained if the input function was replaced by a known function, such as a scaled gamma function, rather than the input function from the measured data.

### 3.2 Image-Based Simulations

In the context of using PET data, the structure of the data is considerably more complex than was used in the 1D function simulations above. In particular, there is considerable spatial correlation in the measured data due to both the inherent underlying biological physiology and the blurring induced by the resolution of the PET camera. Theoretically, weak dependence of this sort is not an issue for FPCA (Hörmann and Kokoszka 2010, 2013). However, from a practical point of view, the performance of the proposed methodology is now assessed in light of these factors.

#### 3.2.1 Simulated Data Generation

To assess the effect of different regions, simulations was performed using a brain phantom (Shepp-Vardi phantom, 128 × 128 pixels) with five different regions of varying sizes (Figure 4). Different signals were placed in each of the regions based on random parameters which also depended on the type of simulation being performed. The level of *V_T_* randomness in each region was about 6.5% roughly equivalent to the voxelwise variability observed within regions in the measured data (Jiang, Aston, and Wang 2009). Finally, the data were blurred using a standard Gaussian blurring kernel, with FWHM of 6 mm, with the voxels in the image being presumed to be 2 mm × 2 mm (as this is a two-dimensional simulation) before time-independent Gaussian errors with variance proportional to the averaged signal were added. The proportionality of this last measurement error results from the quasi-Poisson nature of errors resulting from Poisson radioactive decay (the reconstruction renders the errors not strictly Poisson, and as such a Gaussian approximation is used in the simulations).

We will compare four deconvolution strategies, the FPCA and spline based (SP) methods from the 1D simulations and an additional two based on PET parametric models. The curve-by-curve method for the 1D simulations was also implemented but found to be considerably worse than either FPCA or SP methods (results not shown), so was not considered further. For completeness, the techniques used are now detailed in full. The first is the standard compartmental model based deconvolution based on first-order linear ODEs. Given the nonnegativity in the parameter values for the model, this can be solved using nonnegative least squares (Lawson and Hanson 1974), known as PET spectral analysis in the PET literature (Cunningham and Jones 1993). This analysis will be performed using the standard software DEPICT. Jiang, Aston, and Wang (2009) showed borrowing spatial information can reduce the noise and thus improve the *V_T_* estimates by PET spectral analysis. Therefore, an additional comparison will be made with spectral analysis after the data have been preprocessed (pDEPICT). Similarly, the approach of O'Sullivan et al. (2009) will also be applied to the presmoothed data (with results being worse if presmoothing is not preformed). Finally, the proposed FPCA methodology will be considered. As *V_T_* is the parameter of interest in the PET study, this will be the target of interest in the simulations.

#### 3.2.2 Image Simulation 1: Compartmental Regions

This first image based simulation was designed to assess the performance of the proposed methodology where a true compartmental structure

$$\begin{array}{c} C_{i}\left(t\right)=\left(\alpha_{i1}e^{\beta_{i1}t}+\alpha_{i2}e^{\beta_{i2}t}\right)⊗I\left(t\right) \end{array}$$

was present everywhere, in this case a two compartmental model. This, of course, favors the DEPICT method, where a compartmental structure is assumed, but given that compartmental models are routinely used in PET analysis, and have proved to be useful models in such cases, it is something that is of interest to assess. The values used in each region, along with its size are given in Table 1, where the parameters, α_*ij*_, β_*ij*_ were chosen to coincide with physiologically plausible parameters values from PET studies.

When comparing the MSE of *V_T_* estimates, Table 2 indicates that the FPCA approach and pDEPICT (DEPICT with presmoothing) outperform standard spectral analysis (DEPICT) in all five regions even though the data are generated from compartment models. pDEPICT performs better in regions 2–4, while FPCA performs better in the rest of the regions. These findings are not too surprising as the data are generated from compartment models which are in favor of the DEPICT approach and Jiang, Aston, and Wang (2009) also has showed borrowing spatial information to reconstruct the signals can further improve the *V_T_* estimates by DEPICT due to noise reduction. However, as can also be seen, a completely nonparametric FPCA approach is still competitive even in this situation, where it is possible to assume the correct model structure. However, as expected, the nonparametric approach (SP) which does not involve FPCs performs very badly due to the high noise levels in a voxelwise analysis. In particular, the SP performance is often reasonable, but occasionally has issues at the boundaries (as was seen in Figure 3), which can yield large values of MSE.

**Table 1:** Parameters for first 2D image simulation

Region	Size	α_*i*1_	α_*i*2_	β_*i*1_	β_*i*2_	*V_T_*
1	9614	–	–	–	–	0
2	5351	0.0060	–	0.0030	–	2.00
3	701	0.0040	0.0023	0.0008	0.0103	4.98
4	14	0.0068	0.0009	0.0007	0.0203	9.24
5	704	0.0007	–	0.0377	–	0.02

#### 3.2.3 Image Simulation 2: Noncompartmental Regions

The purpose of this second simulation is to investigate how these four approaches perform when the IRFs are not generated from compartment models. Again, we use the brain phantom image with five different regions; however, we replace the IRFs in regions 2 and 4 with scaled survival functions while the other three regions remain the same, thus incorporating a mixture of both compartmental and noncompartmental regions in the simulation. The level of *V_T_* randomness in these two regions is again taken to be are around 6.5%. The blurring procedure in the final step is identical to simulation 1. Here is the scaled survival function for region 2,

(15) $$\begin{array}{c} M_{i}\left(t\right)=\frac{1}{200}\left[1-\int_{0}^{t}\left\{0.7f_{i1}\left(u/60\right)+0.3f_{i2}\left(u/60\right)\right\}du\right], \end{array}$$

where *f*_*i*1_(*t*) and *f*_*i*2_(*t*) are gamma probability density functions (pdf's) with parameters (α_*i*1_, β_*i*1_) and (α_*i*2_, β_*i*2_) and α_*i*1_ ∼ *N*(1.5, 0.05^2^), α_*i*2_ ∼ *N*(10, 0.5^2^), β_*i*1_ ∼ *N*(2, 0.2^2^) and β_*i*2_ ∼ *N*(1.5, 0.1^2^). The fraction $\frac{1}{200}$ is to make the integral of *M_i_*(*t*) close to a real value ( ≈ 1.97). In region 4, the scaled survival function is

(16) $$\begin{array}{c} M_{i}\left(t\right)=\frac{1}{70}\left[1-\int_{0}^{t}\left\{0.8f_{i1}\left(u/60\right)+0.2f_{i2}\left(u/60\right)\right\}du\right], \end{array}$$

where *f*_*i*1_(*t*) and *f*_*i*2_(*t*) are gamma pdf's with parameters (α_*i*1_, β_*i*1_) and (α_*i*2_, β_*i*2_) and α_*i*1_ ∼ *N*(2, 0.15^2^), α_*i*2_ ∼ *N*(15, 0.1^2^), β_*i*1_ ∼ *N*(2.5, 0.2^2^) and β_*i*2_ ∼ *N*(2, 0.15^2^). The fraction $\frac{1}{70}$ is to make the integral of *M_i_*(*t*) close to a real value ( ≈ 8.54). The parameters are provided in Table 3. The IRF of region 4 has a marked deviation from a compartmental (sum of exponential) structure, while region 2 much more closely resembles a traditional exponential decay, even though it is in fact not expressible as such.

**Table 2 T0002:** Averaged MSE (s.e.) of *V_T_*'s based on 50 runs for five different regions in first 2D simulation

Region	1	2	3	4	5
FPCA	0.0114	0.0832	0.4943	0.6228	0.0433
	(0.0012)	(0.0109)	(0.1471)	(0.8306)	(0.0076)
DEPICT	0.1306	0.2204	0.5549	0.6730	0.1741
	(0.0113)	(0.0076)	(0.0507)	(0.4069)	(0.0149)
pDEPICT	0.0248	0.0601	0.2335	0.2594	0.0505
	(0.0033)	(0.0049)	(0.0481)	(0.2238)	(0.0086)
SP	0.0155	1.1710	7.7870	19.5207	0.2936
	(0.0014)	(0.0849)	(0.4739)	(4.4023)	0.0424

Table 4 shows the MSE of *V_T_* estimates of the three approaches. As in simulation 1, the FPCA approach outperforms DEPICT in all five regions. pDEPICT outperforms DEPICT except in region 4 and the FPCA approach outperforms pDEPICT in regions 1, 4, and 5. This simulation shows that the preprocessing procedure carried out in pDEPICT can help the DEPICT approach to improve the *V_T_* estimates when compartmental conditions are satisfied; however, it does not always work well. If the true IRFs are close to the assumed compartmental structure, as in region 2, the gains from a model based deconvolution can outweigh the model misspecification errors. However, in situations, such as in region 4, where the true IRF is markedly different from a compartmental structure, borrowing spatial information from neighboring voxels can result in worse estimates not only against the nonparametric FPCA deconvolution, but even against the standard DEPICT result where no spatial information is taken into account. On the contrary, the FPCA approach estimates the IRFs nonparametrically and thus the performance is more robust and relatively stable regardless of the model structure. Again, a voxelwise nonparametric deconvolution strategy is not competitive, with SP again suffering from large discrepancies in a few of the simulation runs, resulting in very large MSE values overall.[Table T0002] [Table T0003] [Table T0004]

**Table 3 T0003:** Parameters for second 2D image simulation

Region	Size	α_*i*1_	α_*i*2_	β_*i*1_	β_*i*2_	*V_T_*
1	9614	–	–	–	–	0.00
2	5351	Equation (3.1)				1.97
3	701	0.0040	0.0023	0.0008	0.0103	4.98
4	14	Equation (3.2)				8.54
5	704	0.0007	–	0.0377	–	0.02

**Table 4 T0004:** Averaged MSE (s.e.) of *V_T_*'s based on 50 runs for five different regions in second 2D simulation

Region	1	2	3	4	5
FPCA	0.0116	0.0968	0.3695	0.7045	0.0429
	(0.0013)	(0.0111)	(0.1293)	(0.4041)	(0.0063)
DEPICT	0.1325	0.2405	0.5545	0.9142	0.1774
	(0.0105)	(0.0106)	(0.0496)	(0.4033)	(0.0170)
pDEPICT	0.0257	0.0747	0.2475	1.1158	0.0529
	(0.0028)	(0.0059)	(0.0491)	(0.5028)	(0.0087)
SP	0.0152	1.1346	7.9536	22.8747	0.2917
	(0.0014)	(0.0894)	(0.5365)	(5.3599)	(0.0471)

**Figure 5:**
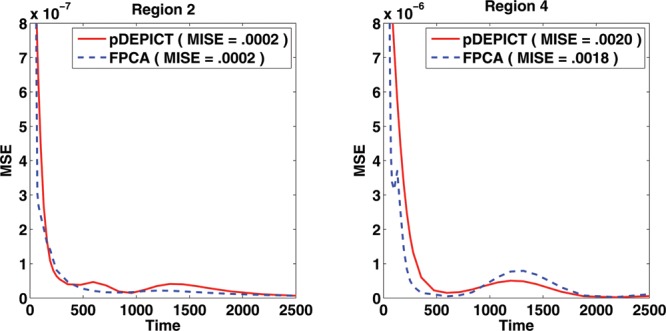
MSE of the different methods in the regions which are not compartmental models in the 2D image simulations.

From the MSE of the estimated functions in region 2 and particularly in region 4, we see that the FPCA approach captures the function shape nicely while DEPICT cannot do so due to its parametric model restrictions. This is emphasized in Figure 5 which examines the pointwise MSE of the reconstructed curves in Regions 2 and 4, as well as the MISE. It should be noted at this point though that simply using MISE as a target in this case would indicate that both approaches perform similarly. However, as *V_T_* is the primary interest, we focused on this, and as can be seen in the Table 4, there is a large improvement in MSE for *V_T_* in Region 4 using FPCA.

**Figure 6:**
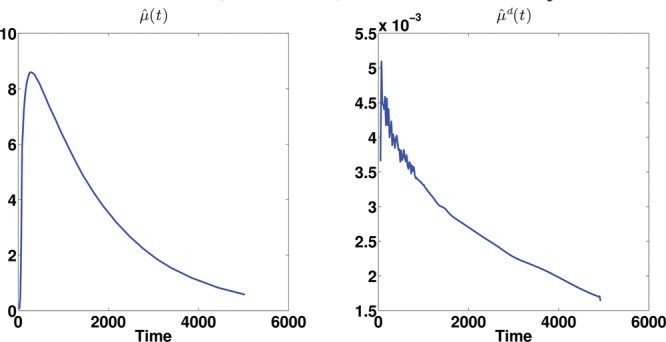
Estimated (deconvolved) mean function for subject 2913.

**Figure 7:**
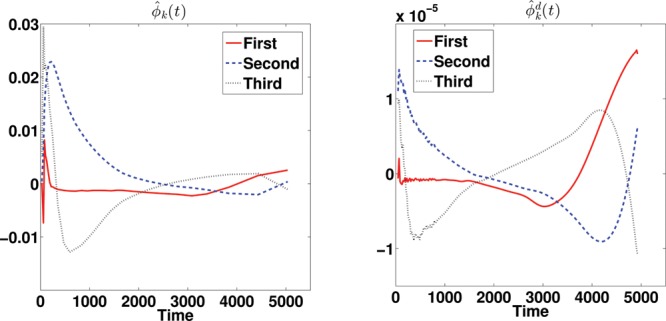
The first three estimated (deconvolved) eigenfunctions for subject 2913.

## 4. MEASURED ^11^C-DIPRENORPHINE DATA

We apply the nonparametric FPCA approach to a set of dynamic PET scans from a measured [^11^C]-diprenorphine study of normal subjects, for which an arterial input function is available. The main purpose of the study is to produce a population of normal controls to build an understanding of opioid receptor densities in normal brain. Multiple subjects were scanned, some once, some twice. We will analyze these data and focus particularly on the repeated scan data, as this analysis will aim to ensure that the methodology is applicable across a range of subjects with reasonable test–retest reproducibility. While reproducibility cannot be equated with the truth, it is somewhat reassuring if the methods yield relatively similar estimates on the same subject in repeated scans. The scans which are analyzed here are part of a study into the relationship between opioid receptors and epilepsy, and the subjects here are from a normal population for the quantification of opioid receptor distribution. In particular, accurate quantification of *V_T_* is required as this is a measure related directly to receptor density. In addition, it is well known that for the tracer [^11^C]-diprenorphine, any particularly compartmental model does not easily fit the data for all voxels (Hammers et al. 2007), so the investigation of a nonparametric approach is of particular relevance here.

The description of the data here follows from Jiang, Aston, and Wang (2009), although in that article only one subject was considered, but the rest were similarly acquired. Each normal control subject underwent either one or two 95-minute dynamic [^11^C]-diprenorphine PET baseline scans. The subject was injected with 185 MBq of [^11^C]-diprenorphine. PET scans were acquired in 3D mode on a Siemens/CTI ECAT EXACT3D PET camera, with a spatial resolution after image reconstruction of approximately 5 mm. Data were reconstructed using the reprojection algorithm (Kinahan and Rogers 1989) with ramp and Colsher filters cutoff at Nyquist frequency. Reconstructed voxel sizes were 2.096 mm × 2.096 mm × 2.43 mm. Acquisition was performed in listmode (event-by-event) and scans were rebinned into 32 time frames of increasing duration. Frame-by-frame movement correction was performed on the dynamic [^11^C]-diprenorphine PET images.

**Figure 8:**
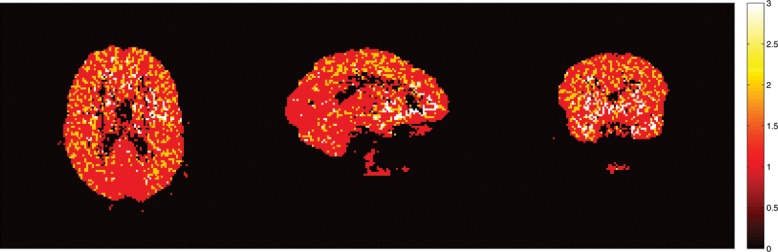
The numbers of components needed to reconstruct the latent signals and the impulse response functions for subject 2913. This indicates that the numbers of components are not randomly distributed in the brain but rather exhibit spatial correlation.

The three most promising approaches used in the simulation studies are applied, DEPICT, pDEPICT, and the nonparametric FPCA procedure, as the data are on the voxel level, and thus curve-by-curve methods are both unstable and computationally intractable so not considered further. We first introduce the results of FPCA on a single subject (no. 2913, who only had one scan, and was randomly chosen for discussion here, although is indicative of the way the analysis performs in general). Figure 6 shows the estimated mean function and its deconvolved function. The deconvolved mean function deviates from the shape which would be expected from a sum of exponential functions. Figure 7 shows the first three eigenfunctions together with their corresponding deconvolved functions. The eigenfunctions indicate the variation from the mean function among voxels.

**Figure 9:**
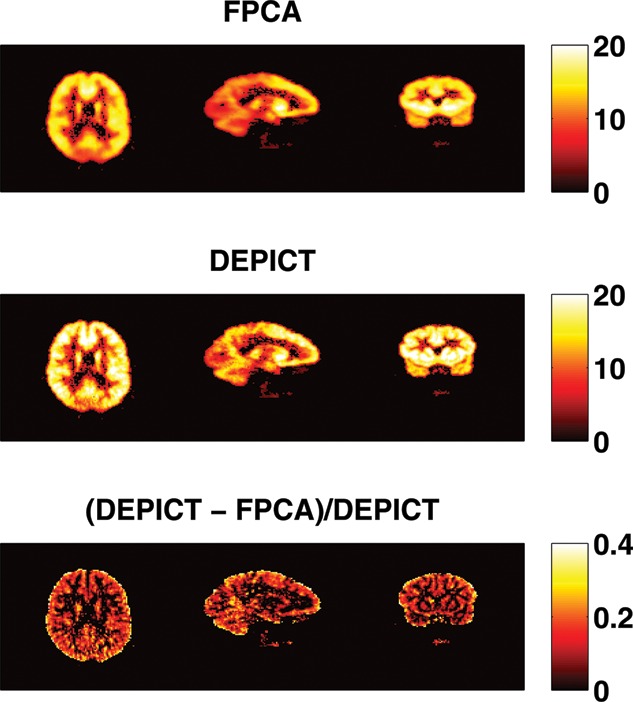
The *V_T_* estimates of FPCA approach and DEPICT approach and their differences for subject 2913. The *V_T_*'s estimated by FPCA are in general smaller than those by DEPICT with *V_T_*'s reduced about 12.4%.

Figure 8 shows the numbers of components needed to reconstruct the latent signals and the impulse response functions. Spatial clusters exist and it gives indications about the concentration of pain receptors in specific spatial locations. Figure 9 shows the *V_T_* estimates of FPCA approach and DEPICT approach and their differences. The *V_T_* estimates by FPCA are about 12.4% lower than those by DEPICT, while pDEPICT was similar to that of DEPICT (approximately 2.2% lower, data not shown). Positive biases of 10% or more are not uncommon for parameter values in the range of those present here (bias is parameter dependent) for PET compartmental models when analyzed with spectral analysis (see Peng et al. 2008), and as such the estimates provided by FPCA are closer to what might be expected from previous simulation results. Thus, the FPCA yields results which are more quantitatively plausible for comparison across a population. In particular, as differences in PET studies between patients and controls tend to be small, and bias is parameter dependent when using nonlinear models, plausible quantitative estimates, which do not rely on particular compartmental assumptions, would allow greater confidence in differences found.

**Figure 10:**
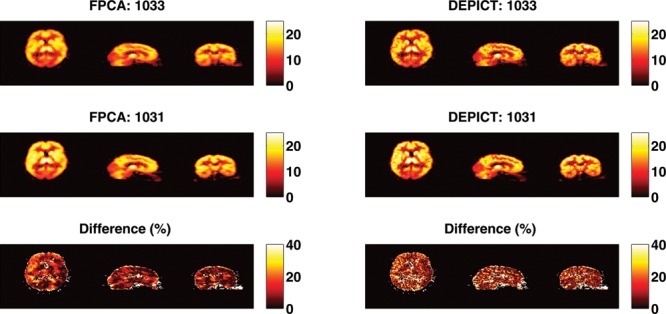
The *V_T_* estimates of a test and a retest scan from a single subject who had two scans (numbered 1031 and 1033). In addition, the percentage change between the two is given for both DEPICT and the FPCA procedure. The difference is truncated at 40% as all voxels above this had estimated *V_T_* close to 0.

The results from the test–retest analysis from the seven subjects who had this data available are given in Table 5 and Figure 10. Taking the figure first, we see that the test–retest variability of both DEPICT and FPCA are roughly similar in corresponding brain regions. This is reassuring as the FPCA procedure is considerably more flexible than the model-based DEPICT estimates. In addition, there is evidence of spatial smoothness in the reproducibility which is physiologically more interpretable from the FPCA approach than the DEPICT approach. Turning to Table 5, we see that there is considerable correspondence between the test–retest results from DEPICT and FPCA. The pooled results show that there are similar levels of variation at different threshold levels. This is important, as the receptor densities are only of interest at somewhat higher *V_T_* values. Indeed there is also less variation within the test–retest values for the FPCA procedure. For individual subjects, the results are fairly balanced with some subjects having smaller test–retest differences with FPCA and others with DEPICT at different threshold levels. However, in almost all cases, the variability in the test–retest results is smaller with FPCA than with DEPICT.

From a computational point of view, the time for the nonparametric deconvolution is very competitive to the parametric modeling approach. The proposed procedure took approximately 8.5 minutes to analyze a single PET scan, which compares with approximately 10 minutes for DEPICT to perform an equivalent analysis (all computations carried out on an Intel core i7 CPU M620 2.67 GHz with 4 GB RAM).

**Table 5:** Averaged absolute normalized difference for [^11^C]-diprenorphine data

	FPCA				DEPICT			
Experiments	5	10	15	20	5	10	15	20
244 vs.247	0.186(0.117)	0.184(0.082)	0.176(0.070)	0.148(0.037)	0.122(0.121)	0.086 (0.070)	0.060(0.047)	0.049(0.036)
1031 vs. 1033	0.131(0.157)	0.088(0.072)	0.073(0.055)	0.055(0.040)	0.158(0.170)	0.110 (0.093)	0.087(0.069)	0.067(0.053)
1248 vs. 1258	0.175(0.111)	0.166(0.056)	0.180(0.041)	0.203(0.034)	0.144(0.120)	0.124 (0.073)	0.147(0.063)	0.163(0.051)
1680 vs. 1774	0.243(0.229)	0.175(0.149)	0.104(0.079)	0.063(0.053)	0.283(0.260)	0.181 (0.154)	0.115(0.111)	0.320(0.354)
1794 vs. 1798	0.238(0.221)	0.184(0.143)	0.170(0.084)	0.262(0.033)	0.295(0.231)	0.260 (0.175)	0.293(0.129)	0.365(0.132)
3427 vs. 3497	0.134(0.130)	0.093(0.064)	0.060(0.041)	NaN	0.166(0.161)	0.106 (0.084)	0.094(0.068)	0.168(0.101)
3568 vs. 3715	0.193(0.183)	0.137(0.110)	0.094(0.077)	0.076(0.062)	0.202(0.191)	0.132 (0.107)	0.095(0.076)	0.099(0.085)
Pooled	0.185(0.175)	0.146(0.110)	0.113(0.078)	0.080(0.067)	0.196(0.196)	0.142(0.127)	0.121(0.109)	0.081 (0.083)

NOTE: We consider the following measure $\frac{|{\hat{V_{T}}}_{1}-{\hat{V_{T}}}_{2}|}{{\hat{V_{T}}}_{2}}$ if ${\hat{V_{T}}}_{2}>\delta$ to evaluate the difference between two experiments. The analysis results from seven individual subjects are pooled and summarized in the last row. Four different δ's (5, 10, 15, and 20) are used.

## 5. DISCUSSION

We have presented a functional data analysis approach to the problem of mass deconvolution in neuroimaging. By expressing the deconvolution problem via a functional principal component basis expansion, it is possible to dramatically reduce the required computational complexity. The methodology has been shown to work well both in generic 1D function deconvolution and also in more realistic image based simulations, while also producing physiologically plausible results in a real data analysis, without resorting to modeling assumptions that are challengeable at best.

The approach to the methodology here has been to take as simple approach as possible for each inherent step. This, of course, could be relaxed, and much more complex algorithms for deconvolution could be investigated in the place of the simple linear deconvolution suggested here. In addition, different methods for choosing the number of eigenfunctions to examine or how the smoothing is performed could also be changed, but without any significant effect on the application of the methodology. However, even the simple approach taken in this article has been shown to be very effective when used in real-data analysis (Zanderigo, Parsey, and Ogden 2015).

It would be possible to carry out a nonparametric analysis using different basis functions using methods such as those explored in O'Sullivan et al. (2014) for FDG. There a segmentation algorithm is used to determine the basis functions and is shown to work well for FDG. However, when using segmentation algorithms, it is often hard to know how many basis functions to use, particularly for tracers such as [^11^C]PK-11195, a marker for neurodegeneration, which has little spatial coherence, and it is not clear that the resulting decomposition would always be identifiable. However, the eigenbasis approach as proposed here would be equally valid in such a situations and by definition always yields an identifiable basis.

We have here suggested the use of the multiplicative random effects model for the FPCA analysis. This could be replaced with the more usual standard FPCA decomposition. However, it has been shown previously (Jiang, Aston, and Wang 2009) that this model is a natural model for PET, given the compartmental assumptions usually made in data modeling, both in terms of its interpretation as well as its empirical performance, and for this reason we have concentrated on it here. It should, of course, be noted that the use of smoothed estimates for the curves, but unsmoothed data to estimate the mean yields the possibility that the functions of the data used to generate the principal component scores will not be eigenfunctions. However, asymptotically (as the smoothing bandwidth goes to zero), these will be consistent estimates. For finite samples, these will still be a completely valid function basis to express the data, albeit not necessarily the finite sample eigenfunctions. However, the gains in using smoothed data to control the noise is considerable over the use of raw curves for deconvolution.

PET volumes are intrinsically three-dimensional tomographic reconstructions. However, the reconstruction process has not been the focus here, but with suitable modification, the methods introduced in this article could be incorporated into reconstruction in a similar way to compartmental analysis (e.g., Wang and Qi 2009), although we have preferred to carry out our analysis in the standard clinical setting directly using the reconstructed data. It is also true that in some PET settings a compartmental model is likely to be a good choice. However, we are advocating using a nonparametric approach due to any particular model being unlikely to be true across the brain for all voxels and indeed model selection for compartmental models is one of the major challenges for practitioners. One drawback of there not being a formal model in the nonparametric case is that *V_T_* does not have the same direct biological interpretation. However, in many cases, the resulting nonparametric *V_T_* will be similar in spirit to that of the compartmental model, and the biological models used to make links from *V_T_* to receptor density, for example, could be updated in line with the finite time nature of the experiment (and after all the experiment really is only performed over a finite time horizon). One interesting line of future research would be to use a mixture approach to the modeling involving both compartmental and nonparametric approaches where some form of model evidence is used to help in the estimation of a common parameter such as *V_T_*.

The methodology presented here is naturally appealing for PET data, given that it reduces the number of deconvolutions from several hundred thousand to four or five. However, it is also a candidate for deconvolution for neuroimaging in general, where in modalities such as fMRI, there is interest in deconvolving hemodynamic response functions from the data (Zhang et al. 2012; Wang et al. 2013). A similar FPCA setup to deconvolve fMRI data could therefore be used, although care would need to be taken and additional regularization used in the deconvolution step, as the null space of the linear operator will be nonzero for fMRI data (due to the negative dip in the hemodynamic response), unlike the case for PET data. Indeed, under suitable assumptions, the approach that has been proposed is applicable in many situations where there are replicates of the curves present, allowing the deconvolution to be treated from a functional data perspective.
